# A prospective study of the association of patient expectations with changes in health-related quality of life outcomes, following total joint replacement

**DOI:** 10.1186/1471-2474-15-248

**Published:** 2014-07-23

**Authors:** Marta Gonzalez Saenz de Tejada, Antonio Escobar, Amaia Bilbao, Carmen Herrera-Espiñeira, Lidia García-Perez, Felipe Aizpuru, Cristina Sarasqueta

**Affiliations:** 1 Research Unit. Red de Investigación en Servicios de Salud en Enfermedades Crónicas (REDISSEC), Basurto University Hospital, Jado, 4th Floor. Avda. Montevideo 18, 48013 Bilbao, Spain; 2 Red de Investigación en Servicios de Salud en Enfermedades Crónicas (REDISSEC), Hospital Universitario Virgen de las Nieves, Avda. de las Fuerzas Armadas, 2, Granada, Spain; 3 Red de Investigación en Servicios de Salud en Enfermedades Crónicas (REDISSEC), Planning and Evaluation Service, Canary Islands Health Service, C/ Pérez de Rozas, n°5, 4th Floor, 38004 Santa Cruz de Tenerife, Spain; 4 Research Unit. Red de Investigación en Servicios de Salud en Enfermedades Crónicas (REDISSEC), Txagorritxu University Hospital, C/ Jose Atxotegi, s/n, 01006 Vitoria-Gasteiz, Spain; 5 Research Unit. Red de Investigación en Servicios de Salud en Enfermedades Crónicas (REDISSEC), Donostia University Hospital, Paseo Dr. Beguiristain s/n. 20014. San Sebastián, Donostia-San Sebastian, Spain

**Keywords:** Expectations, Satisfaction, Osteoarthritis, Total joint replacement

## Abstract

**Background:**

Patient expectations regarding surgery may be related to outcomes in total joint replacement (TJR). The aim of this study was to determine the association of patient expectations with health related quality of life (HRQoL) outcomes measured by Western Ontario and McMaster Universities Osteoarthritis Index (WOMAC) and Short Form 12 (SF-12) and satisfaction with current symptoms measured on a 4-point Likert scale, one year after surgery, adjusting for Body Mass Index (BMI), age, gender, joint, education, previous intervention and baseline scores.

**Methods:**

Consecutive patients preparing for TJR of the knee or hip due to primary osteoarthritis (OA) in 15 hospitals in Spain were recruited for the study. Patients completed questionnaires before surgery and 12 months afterwards: five questions about expectations before surgery; an item to measure satisfaction; two HRQoL instruments—WOMAC and SF-12; as well as questions about sociodemographic information. To determine the association of patient expectations at baseline, with changes in HRQoL 12 months after surgery and with satisfaction, general linear models and logistic regression analysis were performed.

**Results:**

A total of 892 patients took part in the study. Patients who had higher pain relief or ability to walk expectations improved more in HRQoL at 12 months. Moreover, patients with high daily activity expectations were more satisfied.

**Conclusions:**

Patients with higher baseline expectations for TJR, improved more in HRQoL at one year and had more likelihood to be satisfied than patients with lower expectations, adjusted for BMI, age, gender, joint, education, previous intervention and HRQoL baseline scores.

## Background

Osteoarthritis (OA) is a chronic degenerative joint disease and a major source of disability in the elderly [1]. The rapid increase in the prevalence of this disease suggests that OA will have a growing impact on health care and public health systems in the near future [2]. Total joint replacement (TJR) for the management of OA is considered to be one of the most cost-effective operations performed [3-5], with well-documented improvements in health related quality of life (HRQoL) and patient benefits, reducing pain and improving physical function [1,6,7].

Nowadays, the impact of patient expectations on outcomes measured by HRQoL is gaining attention [8-10]. Patient expectations have generally been defined in terms of desires, needs, or requests [11]. Other definitions differentiate between expectations and desires, such as the definition by Uhlmann et al. which describes patient expectations as anticipation that given events are likely to occur during, or as a result of, medical care, in contrast to patient desires, which reflect the patient’s wishes that a given event occur [8,9]. Following the expectation’ definitions carried out by Haanstra et al. [12] for this study, expectations are defined as outcome expectations: beliefs that certain actions will achieve particular outcomes. One reason for the growing interest in the relationship between expectations and HRQoL outcomes after TJR is that psychological factors in patients, such as expectations of outcome, have been found to be important contributors to the success of rehabilitation [13] and are linked to levels of postoperative pain and functional recovery [14,15].

Some researches explain that patient expectations are strongly associated with the physician’s expectations. For TJR surgery, health professionals can play important roles in positively influencing patient expectations. Providing appropriate expectations for patients helps them to develop attainable aims about their recovery and the support strategies to achieve it [16].

The study and measurement of patients’ expectations are necessary to provide more focused clinical care, highlight areas for patient education and promote shared decision-making when several treatment options are available [17]. With regard to treatment outcomes, patient expectations are important considerations for orthopedic surgery, particularly for elective procedures such as TJR [18]. Moreover, preoperative patients’ expectations are potentially important determinants of clinical outcomes and satisfaction [8,19]. Several studies have shown that patients have multiple expectations about hip, knee, back, and shoulder arthroplasty that encompass symptom relief, improvement in physical function, and improvement in psychological well-being [10,20]. In addition, OA patients with high expectations for the benefits of TJR and those who fulfilled expectations, have greater gains in HRQoL and are associated with higher satisfaction with the surgery results [10,21].

Patients’ satisfaction is an important measure of outcome for a variety of reasons [22,23]. Satisfaction has been related to increased patient compliance [24] and patients who are satisfied also tend to return for follow-up care and monitoring [21,24]. A number of patients are not entirely satisfied with the surgery results [25,26]. This may be because the patients’ satisfaction with the outcome of a TJR is a complex concept and is affected by many factors such as incomplete relief from pain, residual functional disability or unmet expectations. Some studies have shown that patients’ expectations were the most important factor influencing patient satisfaction [27,28].

For any procedure and particularly for those that are widely utilized, validated tools should be used to determine the success of the procedure from the patient’s perspective. The HRQoL instruments most utilized in OA had been the generic instruments Medical Outcomes Study Short Form 36 (SF-36) and Medical Outcomes Study Short Form 12 (SF-12) and the disease-specific, Western Ontario and McMaster Universities Osteoarthritis Index (WOMAC) [29].

Previously indicated studies pointed out the important association of baseline expectation with surgical outcomes and satisfaction [8,19]. Nevertheless, these investigations in joint replacement have inconclusive findings, in part due to the retrospective nature of the studies or failure to use multivariable models to identify the relative importance of predictors [8,21,30-32]. In a recent systematic review, Haanstra et al. showed that in general there is limited evidence for an association between patient expectations and treatment outcomes in TJR and highlighted the need for more research in this area [12].

The objective of this study was to determine the association of baseline patient expectations with change on HRQoL outcomes measured by WOMAC and SF-12, and with satisfaction with current symptoms measured on a 4-point Likert scale, at one year post-intervention, adjusting for confounding variables such as, joint, gender, age, education, Body Mass Index (BMI), previous intervention and baseline HRQoL scores.

## Methods

This study was conducted in 15 hospitals in three Spanish regions: three in Andalusia, three in the Canary Islands, and nine in the Basque Country. The institutional review board of the Basurto University Hospital in Bilbao approved the study, code PI04/0938 (September 15, 2004). Written informed consent was obtained from participant patients. Our research has adhered to the STROBE guidelines [33].

Consecutive patients, scheduled to undergo TJR because of primary knee or hip OA in one of the hospitals between October 2005 and October 2006 and who received postoperative management in the participant hospitals, were eligible for the study. Patients with cancer or severe organic or psychiatric diseases were excluded because these conditions could prevent them from completing all the questionnaires included in the study. Besides patients underwent of revision TJR were excluded. All patients were sent a letter informing them about the study and asking for their voluntary participation. We mailed questionnaires to each patient at baseline. The mean time that patients waited since they responded to were underwent was 21.20 days (standard deviation (SD), 42.77). Reminder letters were sent 15 days after each mailing to patients who had not replied promptly. The baseline questionnaire included items about expectations and about HRQoL measured by the SF-12 and WOMAC questionnaires, plus questions requesting sociodemographic information such as gender, age, affected joint, education level, previous intervention and weight and height (for the calculation of BMI). The 12-month mailing included the SF-12 and WOMAC instruments and one question about satisfaction. The data used in this study comprise a subset of patients who have completed preoperative and postoperative HRQoL questionnaires, expectations and satisfaction items.

SF-12 is a generic instrument for measuring HRQoL [34]. Scores for the SF-12 scales range from 0 to 100, where a higher score indicates better health status. There are two summary scores: the physical component summary (PCS) and the mental component summary (MCS). The SF-12 has been translated and validated in Spanish populations, and the measurement properties were published elsewhere [35].

WOMAC is a disease-specific, self-administered questionnaire developed to study patients with hip or knee OA [29]. It has a multidimensional scale made up of 24 items grouped into three dimensions: pain (5 items), stiffness (2 items), and physical function (17 items). Scores range from 0 (none) to 4 (extreme). The data were standardized to a range of values from 0 to 100, where 0 represents the best health status and 100 the worst. The WOMAC has been translated and validated into Spanish [36,37].

Questions in the baseline survey regarding patients’ preoperative expectations for TJR covered five main areas taken from Mancuso’s instrument [21,38]: pain relief, improved ability to perform daily activities, improved ability to walk, improved ability to interact with others, and improved psychological well-being. Our version is an adaptation of Mancuso’s instrument because this author asked about how important expectations were in the treatment for OA; however we asked about how many expectations they have on treatment outcomes because this was more closely adjusted with our environment. Example: "How many expectations do you have to improve ability to walk?" Responses were graded on a five-point Likert scale: no expectations; low expectations; moderate expectations; high expectations, and very high expectations. Responses to the preintervention questions about expectations were highly skewed, so we combined the three lowest groups ("no expectations", "low expectations", and "moderate expectations") into a "low expectations" group. For example, the response distribution for the preintervention expectation of ability to walk was 0.1% for "no expectations", 1.5% for "low expectations", 7.8% for "moderate expectations", 40.4% for "high expectations", and 50.2% for "very high expectations". These responses were categorized into three groups with the following distribution: low expectations, 9.4%; high expectations, 40.4%; and very high expectations, 50.2%. The remaining items followed a similar distribution.

Patient satisfaction was assessed 12 months after surgery with a single question asking "If you had to be the rest of your life with the symptoms you have now, how would you feel?". As we have seen in the literature [21,39,40], most of the patients were satisfied one year after the intervention, so responses of "very dissatisfied" and "somewhat dissatisfied" were collapsed into a dissatisfied group and responses of "very satisfied" and "somewhat satisfied" were collapsed into a satisfied group.

### Statistical methods

Descriptive data are expressed as frequency and percentages, and means with SD. SF-12 changes were calculated by the difference between 12 months scores and baseline scores, and a reverse procedure for WOMAC, with a positive result indicating a gain in HRQoL. We used the *t* test or the nonparametric Wilcoxon test for quantitative variables and chi-square test or the Fisher’s exact test for qualitative variables to assess the differences in baseline patients’ characteristics between responders and non-responders at 12 months.

To determine the association of patient level of expectations at baseline with changes in HRQoL 12 months after surgery, general linear models (GLM) were performed. First, we studied the association of each of the patient expectation items with changes in HRQoL in individual analyses considering the change in HRQoL 12 months after surgery as dependent variable, and each patient expectation as independent variables. Because of the importance of the baseline scores in change in HRQoL [41,42] we have adjusted for the corresponding baseline HRQoL scores. Then, we determined the association of baseline patient expectations jointly with changes in HRQoL 12 months after surgery, adjusting for the corresponding baseline HRQoL scores and possible confounding variables. Change in HRQoL was considered as a dependent variable, and patient expectations, baseline HRQoL scores and confounding variables, joint, gender, age, education, BMI, and previous intervention were considered as independent. The interaction between each patient expectation item and the joint were also considered. Only variables with a statistically significant results remained in the final models. The results of the GLM are reported as beta parameter, which represents the improvement on changes in HRQoL for each unit increase in the independent variable, if it is continuous. If the covariate is categorical, the beta parameter represents the difference in changes in HRQoL of a category with respect to the reference category. The predictive accuracy for each final model was determined by the R^2^. Further, multilevel analysis with mixed models was also performed to test whether the effect of the participating hospital changed the results of the final adjusted models.

In addition, after determining the baseline patient expectations which were associated with changes in WOMAC domains 12 months after surgery in the multivariate models, with the aim to measure whether the observed associations were important in size we compared the minimal clinically important difference (MCID) proportion according to expectation categories ("low", "high" and "very high" expectation). The MCID has been defined as the smallest difference between the scores in a questionnaire that the patient perceives to be beneficial [43]. For this purpose, we classified patients according to the cut-off points of the MCID established for this type of procedure [44,45]. Then, we estimated the MCID proportion (MCID%), which is the proportion of the sample with a change in scores exceeding the MCID, and we compared the MCID% according to expectation categories.

To analyze the association of patient level of expectations at baseline with patients’ satisfaction 12 months after surgery, logistic regression analysis was performed, following the same steps as before. First, we studied the association of each patient expectation individually with satisfaction 12 months after surgery considering satisfaction as a dependent variable, and each patient expectation as independent variables. Then, we determined the association of baseline patient expectations jointly with satisfaction 12 months after surgery, adjusting for possible confounding variables. Satisfaction was considered as a dependent variable, and patient expectations, and confounding variables such as, joint, gender, age, education, BMI, and previous intervention were considered as independent. The interaction between each patient’s expectations and joint were also considered. Only those variables with a statistically significant result were remained in the final models. The results of the models are reported as odds ratios (OR) and 95% confidence interval (CI). The predictive accuracy for the final model was determined by the area under the receiver operating characteristic (ROC) curve (AUC) [46]. Furthermore, multilevel analysis with generalized estimated equations was also carried out to determine if the effect of the participating hospital changed the results of the final adjusted models.

All effects were considered statistically significant at *p* < 0.05. All statistical analyses were performed using SPSS (SPSS Inc., Chicago, IL) version 18.0, and SAS for Windows statistical software, version 9.2 (SAS Institute Inc., Cary, NC).

## Results

A total of 1681 patients on waiting lists for TJR who fulfilled the inclusion criteria and were not excluded by the exclusion criteria agreed to participate in the study and completed the baseline questionnaire before surgery. After the intervention, 892 (53.6%) completed the follow-up questionnaire at 12 months. This is the sample included in the study. The mean age was 68.74 years (SD = 9.92), 59.37% were women, 40.92% underwent total hip replacement, 63.6% had not had a previous intervention, the mean BMI was 29.37 (SD = 4.61) and 59.21% had primary education. Baseline SF-12 and WOMAC HRQoL data, as well as a comparison with the data from non-responders, are included in Table 1. Nonresponders had slightly worse scores in the three WOMAC domains and in the MCS SF-12 domain than responders. In expectation items there were baseline statistically significant differences between responders and non-responders. Responders had higher expectations than non-responders.

**Table 1:** Baseline patients’ characteristics of responders and non-responders to the 12-month follow-up questionnaire

	**Responders (N = 892)**	**Non-responders (N = 789)**	***p value***
Age in years: mean (SD)	68.74 (9.92)	69.07 (9.65)	0.505
Joint: hip: n (%)	365 (40.92%)	289 (36.26%)	0.051
Gender: female: n (%)	529 (59.37%)	507 (63.77%)	0.71
BMI: mean (SD)	29.37 (4.61)	29.98 (4.71)	0.012
WOMAC: mean (SD)			
Pain	55.26 (18.17)	58.60 (19.54)	<0.001
Stiffness	56.92 (24.24)	60.70 (24.46)	0.002
Function	62.53 (17.28)	66.65 (17.28)	<0.001
SF-12: mean (SD)			
PCS	29.80 (7.08)	29.69 (7.46)	0.787
MCS	43.34 (14.23)	41.21 (14.40)	0.006
Higher education: n (%)			
Secondary education/University	176 (20.39%)	121 (15.71%)	<0.001
Primary education	511 (59.21%)	419 (54.42%)	
None	176 (20.39%)	230 (29.87%)	
Previous intervention: n (%)			
Yes	311 (36.4%)	287 (37.18%)	0.758
No	543 (63.6%)	485 (62.82%)	
Expectation items			
Pain relief: n (%)			
Low	89 (10.31%)	135 (17.26%)	<0.001
High	287 (33.26%)	282 (36.06%)	
Very high	487 (56.43%)	365 (46.68%)	
Daily activities: n (%)			
Low	165 (18.94%)	215 (27.53%)	<0.001
High	407 (46.73%)	326 (41.74% )	
Very high	299 (34.33%)	240 (30.73%)	
Ability to walk: n (%)			
Low	82 (9.40%)	130 (16.71%)	<0.001
High	352 (40.37%)	329 (42.29%)	
Very high	438 (50.23%)	319 (41.00%)	
To interact with others: n (%)			
Low	221 (26.06%)	257 (33.68%)	0.002
High	330 (38.92%)	330 (38.92%)	
Very high	297 (35.02%)	221 (28.96%)	
Psychological well-being: n (%)			
Low	171 (20.38%)	200 (26.49%)	0.015
High	355 (42.31%)	290 (38.41%)	
Very high	313 (37.31%)	265 (35.10%)	

Score direction: WOMAC, higher scores indicating worse HRQoL; SF-12, higher scores indicating better HRQoL.

SD, Standard deviation; BMI, Body Mass Index; WOMAC, Western Ontario and McMaster Universities Osteoarthritis Index; SF-12, Short Form 12; PCS, physical component summary of the SF-12; MCS, mental component summary of the SF-12.

Patients’ preoperative expectations were quite high (Table 1). If we take into account the "high expectations" and "very high expectations", the areas in which patients had the highest expectations of improvement were ability to walk after surgery, and improved pain relief, with 90.6% and 89.7%, respectively. They were followed closely by doing more daily activities (81.1%), improved psychological well-being (79.6%), and improved capacity to interact with others (73.9%).

### Patients’ expectations and change in HRQoL

Regarding associations between each of the expectation questions and change in HRQoL from baseline to 12 months (Table 2), patients’ expectations showed, in general, a statistically significant association in all HRQoL domains, so, the higher patients’ expectations, the more they improved. Therefore, patients with high or very high pain relief or daily activities expectations improved more in all HRQoL domains except SF-12 MCS domain, than patients with low expectations. With regard to ability to walk, interact with others and psychological wellbeing expectations, patients with very high expectations showed more improvement in all WOMAC domains than patients with low expectations, and patients with high or very high expectations showed more improvement in SF-12 PCS domain than patients with low expectations.

**Table 2:** Association of patient expectations individually with change in HRQoL at 12 months by general linear models (N = 892)

**Expectations**	**Change in WOMAC**						**Change in SF-12**			
	**Pain**		**Stiffness**		**Function**		**PCS**		**MCS**	
	**β**	***p value***	**β**	***p value***	**β**	***p value***	**β**	***p value***	**β**	***p value***
Pain Relief										
Low	Ref.	—	Ref.	—	Ref.	—	Ref.	—	Ref.	—
High	11.29	<0.001	9.41	0.012	9.10	0.001	6.30	0.001	4.51	0.066
Very high	14.43	<0.001	15.64	<0.001	12.10	<0.001	9.46	<0.001	4.584	0.049
Daily activities										
Low	Ref.	—	Ref.	—	Ref.	—	Ref.	—	Ref.	—
High	5.47	0.012	6.96	0.015	5.13	0.017	3.82	0.008	0.35	0.849
Very high	8.11	<0.001	10.95	<0.001	7.37	0.001	7.79	<0.001	0.10	0.957
Ability to walk										
Low	Ref.	—	Ref.	—	Ref.	—	Ref.	—	Ref.	—
High	3.41	0.235	4.21	0.267	4.28	0.129	4.53	0.017	1.58	0.515
Very high	10.90	<0.001	13.87	<0.001	11.01	<0.001	9.50	<0.001	4.08	0.084
Interact with others										
Low	Ref.	—	Ref.	—	Ref.	—	Ref.	—	Ref.	—
High	4.02	0.050	1.21	0.651	3.88	0.055	3.29	0.013	2.62	0.116
Very high	5.31	0.012	8.82	0.001	6.74	0.001	6.20	<0.001	0.58	0.726
Psychological well-being										
Low	Ref.	—	Ref.	—	Ref.	—	Ref.	—	Ref.	—
High	2.97	0.176	0.55	0.848	1.75	0.419	3.68	0.011	0.70	0.701
Very high	8.39	<0.001	10.17	0.001	8.33	<0.001	6.67	<0.001	3.09	0.093

Ref: reference group.

This general linear models have been adjusted for the corresponding HRQoL baseline scores.

WOMAC, Westen Ontario and McMaster Universities Osteoarthritis Index; SF-12, Short Form 12; PCS, physical component summary of the SF-12; MCS, mental component summary of the SF-12.

Changes are calculated so that a positive value indicates improvement and a negative value, worsening in all questionnaires.

Table 3 shows the results of multivariate general linear models to determine the association of baseline patient expectations jointly with changes in WOMAC and SF-12 domains 12 months after surgery, adjusting for confounding covariables and baseline HRQoL scores. Patients with higher expectations were associated with higher improvements in HRQoL at 12 months. There were two the expectations associated regarding WOMAC pain and function domains: patients with high or very high pain relief expectations improved more than patients with low expectations and patients with very high ability to walk expectations improved more than patients with low or high expectations. Expectations associated with WOMAC stiffness were pain relief. Regarding SF-12 PCS domain patients with very high ability to walk expectations were associated with more improvements than patients with low or high expectations. Finally patients with high or very high pain relief expectations improved more in SF-12 MCS than those with low expectations. The covariables that showed association with change in HRQoL at 12 months were baseline HRQoL scores, joint, BMI, education and previous intervention. Explanatory ability of the models, in the case of change in WOMAC and SF-12 domains at 12 months, were from 35% to 57%, apart from SF-12 PCS (R^2^ = 0.24). Multilevel analysis showed that the previous results remained after adjusting for the effect of the participating hospital.

**Table 3:** Association of patients expectations jointly with changes in HRQoL at 12 months adjusting for baseline HRQoL scores and other covariables by general linear models (n = 892)

**Covariables and expectations**	**Changes in WOMAC**						**Changes in SF-12**			
	**Pain**		**Stiffness**		**Function**		**PCS**		**MCS**	
	**β**	***p value***	**β**	***p value***	**β**	***p value***	**β**	***p value***	**β**	***p value***
**Baseline HRQoL**	0.82	<0.0001	0.94	<0.0001	0.72	<0.0001	-0.75	<0.0001	-0.69	<0.0001
**BMI**							-0.25	0.0175		
**Joint**										
Knee	Ref.	—	Ref.	—	Ref.	—				
Hip	4.16	0.0013	5.65	<0.0001	3.24	0.0207				
**Highest degree**										
Secondary education/ University	6.60	0.0011					3.91	0.0011		
Primary education	3.09	0.0517					^*^Ref.	—		
None	Ref.	—								
**Previous intervention**										
Yes					-3.91	0.0064				
No					Ref.	—				
**Expectations**										
**Pain Relief**										
Low	Ref.	—	Ref.	—	Ref.	—			Ref.	—
High	10.52	<0.0001	6.63	0.0079	11.14	<0.0001			4.96	0.0081
Very high	10.22	<0.0001	7.95	0.0009	11.99	<0.0001			6.97	<0.0001
**Ability to walk**										
Low or High	Ref.	—			Ref.	—	Ref.	—		
Very high	3.32	0.0370			3.78	0.0248	3.89	<0.0001		
**R**^**2**^	0.45		0.57		0.35		0.24		0.42	

Ref: reference group.

*In this case there has been grouped the reference group (primary and none education).

Baseline Score direction: WOMAC, higher scores indicating worse HRQoL; SF-12, higher scores indicating better HRQoL.

BMI, Body Mass Index; WOMAC, Westen Ontario and McMaster Universities Osteoarthritis Index; SF-12, Short Form 12; PCS, physical component summary of the SF-12; MCS, mental component summary of the SF-12. Ref: reference group.

Changes are calculated so that a positive value indicates improvement and a negative value, worsening in all questionnaires.

Taking into account that after adjustments, the two expectations which were associated with change in WOMAC domains were those related to pain relief and ability to walk, we compared the MCID% for each WOMAC change score according to expectation categories. Among patients with low pain relief expectations, the patients exceeding the MCID in WOMAC domains after surgery varied from 53.4% to 58.4%, while this range was higher for patients with high or very high expectations, which varied from 75.0% to 79.2%, except for WOMAC stiffness domain which was 62.8% and 69.2% for high and very high expectations, respectively. Regarding ability to walk expectations, the percentage of patients with low expectations exceeding the MCID after surgery ranged from 58.2% to 64.2%, whereas this range was higher for patients with high or very high expectations, which varied from 70.7% to 81.0%, apart from WOMAC stiffness domain with a percentage of 61.1%.

### Expectation and Satisfaction

In Table 4 we can observe the unadjusted association of level of expectations with satisfaction 12 months after surgery. In the univariate logistic model expectations related to satisfaction were pain relief, daily activities and ability to walk, but in the multivariate logistic model only daily activities expectations remained significantly associated with satisfaction after surgery. So, we do not show results of the multivariate logistic model because these are the same that appeared in the univariate logistic model. Thus, patients with high or very high daily activities expectations had more likelihood to be satisfied than patients with low expectations. No adjusting covariable was associated with satisfaction at 12 months in the multivariate logistic model. The AUC of the model was 0.57. Multilevel analysis showed that the previous results remained with the adjustment for the effect of the participating hospital.

**Table 4:** Univariate logistic regression analysis for the association of patients’ expectations on satisfaction at 12 months (n = 892)

**Expectations**	**Satisfaction (univariate)**	
	**Odds ratio (95% CI)**	***p value***
**Pain relief**		
Low	Ref.	—
High	2.31 (1.34 – 4)	0.003
Very high	2.29 (1.37 – 3.80)	0.001
**Daily activities**		
Low	Ref.	—
High	1.73 (1.13 – 2.65)	0.012
Very high	2.21 (1.38 – 3.54)	0.001
**Ability to walk**		
Low	Ref.	—
High	1.60 (0.92 – 2.80)	0.099
Very high	1.96 (1.13 – 3.41)	0.018
**Interact with others**		
Low	Ref.	—
High	1.38 (0.91 – 2.11)	0.132
Very high	1.55 (1 – 2.40)	0.052
**Psychological well-being**		
Low	Ref.	—
High	1.22 (0.77 – 1.91)	0.397
Very high	1.34 (0.84 – 1.24)	0.222

Ref: reference group;

CI: Confidence interval.

## Discussion

Our prospective study of a sample of consecutive patients with knee or hip OA undergoing TJR offers insight into the association of patient expectations with HRQoL outcomes and satisfaction at one year post-intervention. We observed that patients with higher expectations improved more in HRQoL, measured by SF-12 and WOMAC questionnaires, and had more likelihood to be satisfied, adjusted for BMI, age, gender, joint, education, previous intervention and HRQoL baseline scores. In spite of the existence of several studies that have measured the association between patients expectations and treatment outcomes in TJR, in general there is limited evidence for this association. Haanstra et al. in a systematic review about it highlighted the need for more research in this field [12]. The strengths of our study include the relatively large sample size, the use of valid and responsive instruments for assessing outcomes in TJR and the use of multivariate analyses adjusting for confounding variables.

Patients in our study had higher expectations for improvements in physical or functional symptoms than in social or psychological capacities. The two areas where patients had the highest expectations were pain relief and ability to walk, which are traditional reasons for performing TJR. Physical and functional expectations are more closely related to the direct effects of the intervention [12]; therefore, patients might look forward to potential benefits that are more closely related to their basal symptoms such as pain or ability to walk.

These expectations were also the ones with the most association with changes in HRQoL at 12 months after surgery, even after adjusting for covariables. This change, besides being statistically significant, was clinically relevant because there was higher percentage of patients who exceeded MCID as expectations increases. Therefore, the areas where patients place more trust in improving, are the ones more associated with change. This could be due to the fact that our patients had realistic expectations of surgery and correctly anticipated the outcome of the intervention. As in previous studies [12,47], our findings show that patients with worse baseline HRQoL are more likely to improve, so high expectations of surgery are realistic, and might explain the association, adjusting for covariables.

Another potential explanation about observed association of greater expectations being related with improved outcomes is that patients with higher preintervention expectations interpreted their gains more optimistically and participated more intensely in their rehabilitation process, which in turn may affect their recovery, as suggested by some studies [8,47].

Keeping this in mind, it would be important to analyse why some patients have low expectations, which could prevent them from improving. It has been found that unrealistically low expectations may not provide the motivation necessary to progress with the recovery, and thus may result in patients not deriving full benefit from TJR [48,49].

There are many studies in TJR about expectations that have pointed out the importance of baseline patients’ realistic expectations so they could be sufficiently fulfilled [10,20,48-50]; however, these studies usually do not emphasize the importance of building up patients’ expectations. So, an alternative point of view is that patients’ higher expectations contributed to outcomes by acting as a psychological factor, which ultimately could have an influence on post-intervention HRQoL [19,51]. Hence as Judge et al. [47] pointed out, it could be argued that surgeons should explain to patients with low expectations that the TJR will be quite successful, building up appropriate expectations.

To our knowledge, there are a few comparable studies that explore the association between pre-operative expectations and change in HRQoL for TJR [12]. In our study we found that all analyzed baseline patient expectations were associated with change in HRQoL 12 months after surgery. However, in our study, like others [8], independent expectations associated with improvement in pain and function domains were pain relief and improved ability to walk, measured by WOMAC and SF-36, after adjusting for covariables. So, patients who have higher pain relief or ability to walk expectations may have perceived less pain, less stiffness, and better functional and mental outcomes than patients with less expectations.

Along the same line, Judge et al. [47] in a study of 1327 primary total hip replacement (THR) explored whether pre-operative expectations predict surgical outcomes in terms of pain and function measured by WOMAC 12 months post THR. They found that the more the preoperative expectations of a patient, the more likely they were to improve at 12 months.

On the other hand, one study of 112 patients who underwent total knee replacement (TKR) [19] examined which was the most important unique determinant of global outcome/satisfaction after surgical management: baseline expectations, fulfillment of expectations or current symptoms and function. Bivariate analyses showed that baseline expectations were associated with change in pain and in functional limitations. Similar outcomes were reported by other studies [8,30]. However, these results did not retain significance in the multivariable model predicting the overall global outcome or satisfaction. A previous study for TKR [52] also found that pain scores were significantly better for patients who had expected to have no pain and/or had expected they would not need a walking aid. However, although it was significant in this large cohort of 598 patients, the magnitude of this difference (5 points) may not be clinically meaningful. Moreover, for TJR a more recent study [20] found that expectations of time to fully recover from surgery and level of function were not predictors of WOMAC change scores. However, having expectations of pain relief was a significant predictor.

Satisfaction is a complex item, which is affected by many factors, especially expectations before the surgery [24]. Like Noble et al. [28] we found that some preoperative expectations were associated with satisfaction after TJR. Having higher pain relief, daily activities and ability to walk expectations seem to be related to more satisfaction than patients with low expectations. However, these differences only persist for daily activities expectations after adjusting for the other covariables. Therefore, in our study, patients who have significant expectations to improve their ability to perform daily activities had more likelihood to be satisfied than patients who had low expectations. Pain and ability to walk could be associated with the ability to perform daily activities. Thus, the less the pain and difficulty in walking the patients have, the greater their ability to perform daily activities; which seems to be associated directly with satisfaction. Patients seem to see more easily the relationship of their ability to perform daily activities with satisfaction, however, this ability could probably be related to pain relief and ability to walk. Following this trend Noble et al. [28] suggest that patient’s expectations will strongly influence their interpretation of the outcome of their TJR and their satisfaction. On the other hand, a US study by Mancuso et al. [21] in 180 THR looked at whether preoperative expectations were associated with satisfaction with surgery. They concluded that expectations were not associated with satisfaction. Finally, it should be noted that we would have to take into account that this relationship between expectations and satisfaction is likely to be mediated by a larger improvement in those with high expectations.

### Limitations

A possible limitation of our study is the percentage of non-responders or missing values. Only 53.06% patients completed questionnaires at 12 months. Probably owed to our questionnaire extension, the patient’s burden to complete the questionnaire could be important. However our sample keeps on being large enough comparing with others similar studies. Besides, TJRs performed in total during the recruitment period are unknown because there were fifteen hospitals of different autonomous community participating and not all of these hospitals did collect this information. As well as owed to large sample size we found differences between responders and non-reponders, nevertheless this differences despite being statistically significant were not clinically relevant, although could cause a bias in the results. Another limitation is that we did not evaluate patients’s knowledge of TJR, coming from their clinical or their social/familiar settings about the procedure, recovery process, complications and so on, as potential covariables associated with functional outcomes and which could strongly influence expectations. Besides, like in other studies [53] in order to estimate the score for satisfaction at one year we used a single anchoring question on patient satisfaction with the surgical outcome. This is a similar concept to widely used Patient Acceptable Symptom State (PASS) [53-57]. The difference is that our item asked about satisfaction with surgery that included aspects of patients’ current symptoms, but also their baseline level of symptoms, in addition to their response and expectations of surgery. Furthermore, we did not use a validated expectation questionnaire that could ensure comparability for future research or an open-ended free text question allowing other types of expectations to be identified. However, these kinds of questions may pose problems regarding how to code answers, and differences in verbosity or fluency could affect the findings [47]. Finally, these questions did not measure the importance of different expectations expressed by individual patients. Therefore future studies need other possible factors that influence expectations.

## Conclusions

In conclusion, this prospective study showed that patients preparing for TJR had high expectations for the surgery, and patients with high or very high baseline expectations for TJR improved more in HRQoL at one year and had more likelihood to be satisfied than patients with low expectations. Given that having high expectations seems to be beneficial to surgical outcomes, surgeons should talk with their patients to providing them with appropriate expectations, which act as a psychological factor that could improve HRQoL outcomes.

## Abbreviations

OA: Osteoarthritis; TJR: Total joint replacement; HRQoL: Health related quality of life; SF-36: Short form 36; SF-12: Short form 12; WOMAC: Westenr Ontario and McMaster Universities Osteoarthritis Index; BMI: Body mass index; SD: Standard deviation; PCS: Physical component summary; MCS: Mental component summary; GLM: General linear models; MCID: Minimal clinically important difference; OR: Odds ratio; CI: Confidence interval; ROC: Receiver operating characteristic; AUC: Area under curve.

## Competing interests

There are no conflicts of interest.

## Authors’ contributions

MG, carried out the acquision of data, involved in design of the study, analysis, interpretation and composition of the manuscript. AE conceived of the study, and participated in its design and coordination and helped to draft the manuscript. AB participated in the draft of the manuscript and performed the statistical analyses. CH, LG, FA, and CS were involved in conception and design of the study. All authors read and approved the final manuscript.

## Pre-publication history

The pre-publication history for this paper can be accessed here:

http://www.biomedcentral.com/1471-2474/15/248/prepub

## References

[B1] Ethgen O Bruyere O Richy F Dardennes C Reginster JY Health-related quality of life in total hip and total knee arthroplasty. A qualitative and systematic review of the literature J Bone Joint Surg Am 2004 86-A 963 974 15118039 10.2106/00004623-200405000-00012

[B2] Lawrence RC Felson DT Helmick CG Arnold LM Choi H Deyo RA Gabriel S Hirsch R Hochberg MC Hunder GG Jordan JM Katz JN Kremers HM Wolfe F Estimates of the prevalence of arthritis and other rheumatic conditions in the United States. Part II Arthritis Rheum 2008 58 26 35 18163497 10.1002/art.23176 PMC3266664

[B3] Rissanen P Aro S Sintonen H Asikainen K Slatis P Paavolainen P Costs and cost-effectiveness in hip and knee replacements. A prospective study Int J Technol Assess Health Care 1997 13 575 588 9489250 10.1017/s0266462300010059

[B4] Krummenauer F Wolf C Gunther KP Kirschner S Clinical benefit and cost effectiveness of total knee arthroplasty in the older patient Eur J Med Res 2009 14 76 84 19258217 10.1186/2047-783X-14-2-76 PMC3351964

[B5] Losina E Walensky RP Kessler CL Emrani PS Reichmann WM Wright EA Holt HL Solomon DH Yelin E Paltiel AD Katz JN Cost-effectiveness of total knee arthroplasty in the United States: patient risk and hospital volume Arch Intern Med 2009 169 1113 1121 19546411 10.1001/archinternmed.2009.136 PMC2731300

[B6] Quintana JM Escobar A Arostegui I Bilbao A Azkarate J Goenaga JI Arenaza JC Health-related quality of life and appropriateness of knee or hip joint replacement Arch Intern Med 2006 166 220 226 16432092 10.1001/archinte.166.2.220

[B7] Bruyere O Ethgen O Neuprez A Zegels B Gillet P Huskin JP Reginster JY Health-related quality of life after total knee or hip replacement for osteoarthritis: a 7-year prospective study Arch Orthop Trauma Surg 2012 132 1583 1587 22842917 10.1007/s00402-012-1583-7

[B8] Mahomed NN Liang MH Cook EF Daltroy LH Fortin PR Fossel AH Katz JN The importance of patient expectations in predicting functional outcomes after total joint arthroplasty J Rheumatol 2002 29 1273 1279 12064846

[B9] Uhlmann RF Inui TS Carter WB Patient requests and expectations. Definitions and clinical applications Med Care 1984 22 681 685 6748787 10.1097/00005650-198407000-00011

[B10] de TM GS Escobar A Herrera C Garcia L Aizpuru F Sarasqueta C Patient expectations and health-related quality of life outcomes following total joint replacement Value Health 2010 13 447 454 20088892 10.1111/j.1524-4733.2009.00685.x

[B11] Cross M Lapsley H Barcenilla A Brooks P March L Patient Expectations of Hip and Knee Joint Replacement Surgery and three months port-operative Health Status 2008 Canberra: Rydges Lakeside

[B12] Haanstra TM van den Berg T Ostelo RW Poolman RW Jansma EP Cuijpers P de Vet HC Systematic review: do patient expectations influence treatment outcomes in total knee and total hip arthroplasty? Health Qual Life Outcomes 2012 10 152 23245187 10.1186/1477-7525-10-152 PMC3568025

[B13] Albrecht GL Higgins PC Rehabilitation success: the interrelationships of multiple criteria J Health Soc Behav 1978 18 36 45 641330

[B14] Taenzer P Melzack R Jeans ME Influence of psychological factors on postoperative pain, mood and analgesic requirements Pain 1986 24 331 342 3960574 10.1016/0304-3959(86)90119-3

[B15] Mondloch MV Cole DC Frank JW Does how you do depend on how you think you’ll do? A systematic review of the evidence for a relation between patients’ recovery expectations and health outcomes CMAJ 2001 165 174 179 11501456 PMC81284

[B16] Frank JD The influence of patients’ and therapists’ expectations on the outcome of psychotherapy Br J Med Psychol 1968 41 349 356 4936798 10.1111/j.2044-8341.1968.tb02043.x

[B17] Tekin B Unver B Karatosun V Expectations in patients with total knee arthroplasty Acta Orthop Traumatol Turc 2012 46 174 180 22659633 10.3944/aott.2012.2655

[B18] Mancuso CA Jout J Salvati EA Sculco TP Fulfillment of patients’ expectations for total hip arthroplasty J Bone Joint Surg Am 2009 91 2073 2078 19723982 10.2106/JBJS.H.01802

[B19] Mannion AF Kampfen S Munzinger U Kramers-de Q I The role of patient expectations in predicting outcome after total knee arthroplasty Arthritis Res Ther 2009 11 R139 19772556 10.1186/ar2811 PMC2787271

[B20] Gandhi R Davey JR Mahomed N Patient expectations predict greater pain relief with joint arthroplasty J Arthroplasty 2009 24 716 721 18701241 10.1016/j.arth.2008.05.016

[B21] Mancuso CA Salvati EA Johanson NA Peterson MG Charlson ME Patients’ expectations and satisfaction with total hip arthroplasty J Arthroplasty 1997 12 387 396 9195314 10.1016/s0883-5403(97)90194-7

[B22] Bullens PH van Loon CJ de Waal Malefijt MC Laan RF Veth RP Patient satisfaction after total knee arthroplasty: a comparison between subjective and objective outcome assessments J Arthroplasty 2001 16 740 747 11547372 10.1054/arth.2001.23922

[B23] Nilsdotter AK Toksvig-Larsen S Roos EM Knee arthroplasty: are patients’ expectations fulfilled? A prospective study of pain and function in 102 patients with 5-year follow-up Acta Orthop 2009 80 55 61 19234886 10.1080/17453670902805007 PMC2823230

[B24] Lochman JE Factors related to patients’ satisfaction with their medical care J Community Health 1983 9 91 109 6678264 10.1007/BF01349873

[B25] Judge A Cooper C Williams S Dreinhoefer K Dieppe P Patient-reported outcomes one year after primary hip replacement in a European Collaborative Cohort Arthritis Care Res (Hoboken ) 2010 62 480 488 20391502 10.1002/acr.20038

[B26] Nilsdotter AK Petersson IF Roos EM Lohmander LS Predictors of patient relevant outcome after total hip replacement for osteoarthritis: a prospective study Ann Rheum Dis 2003 62 923 930 12972468 10.1136/ard.62.10.923 PMC1754324

[B27] Brokelman R Van LC Van SJ Van KA Veth R Patients are more satisfied than they expected after joint arthroplasty Acta Orthop Belg 2008 74 59 63 18411603

[B28] Noble PC Conditt MA Cook KF Mathis KB The john insall award: patient expectations affect satisfaction with total knee arthroplasty Clin Orthop Relat Res 2006 452 35 43 16967035 10.1097/01.blo.0000238825.63648.1e

[B29] Bellamy N Buchanan WW Goldsmith CH Campbell J Stitt LW Validation study of WOMAC: a health status instrument for measuring clinically important patient relevant outcomes to antirheumatic drug therapy in patients with osteoarthritis of the hip or knee J Rheumatol 1988 15 1833 1840 3068365

[B30] Engel C Hamilton NA Potter PT Zautra AJ Impact of two types of expectancy on recovery from total knee replacement surgery (TKR) in adults with osteoarthritis Behav Med 2004 30 113 123 15816314 10.3200/BMED.30.3.113-123

[B31] Burton KE Wright V Richards J Patients’ expectations in relation to outcome of total hip replacment surgery Ann Rheum Dis 1979 38 471 474 518147 10.1136/ard.38.5.471 PMC1000396

[B32] Moran M Khan A Sochart DH Andrew G Expect the best, prepare for the worst: surgeon and patient expectation of the outcome of primary total hip and knee replacement Ann R Coll Surg Engl 2003 85 204 206 12831497 10.1308/003588403321661415 PMC1964367

[B33] Von EE Altman DG Egger M Pocock SJ Gotzsche PC Vandenbroucke JP The Strengthening the Reporting of Observational Studies in Epidemiology (STROBE) statement: guidelines for reporting observational studies J Clin Epidemiol 2008 61 344 349 18313558 10.1016/j.jclinepi.2007.11.008

[B34] Ware J Jr Kosinski MM Keller SDP SDP: A 12-Item Short-Form Health Survey: construction of scales and preliminary tests of reliability and validity Med Care 1996 34 3 220 233 8628042 10.1097/00005650-199603000-00003

[B35] Gandek B Ware JE Aaronson NK Apolone G Bjorner JB Brazier JE Bullinger M Kaasa S Leplege A Prieto L Sullivan M Cross-validation of item selection and scoring for the SF-12 Health Survey in nine countries: results from the IQOLA Project. International Quality of Life Assessment J Clin Epidemiol 1998 51 1171 1178 9817135 10.1016/s0895-4356(98)00109-7

[B36] Batlle Gualda E Esteve Vives J Piera Riera M Hargreaves R Cutts J Traducción y adaptación al español del cuestionario WOMAC específico para artrosis de rodilla y cadera Rev Esp Reumatol 1999 26 2 38 45 35

[B37] Escobar A Quintana JM Bilbao A Azkarate J Guenaga JI Validation of the Spanish version of the WOMAC questionnaire for patients with hip or knee osteoarthritis. Western Ontario and McMaster Universities Osteoarthritis Index Clin Rheumatol 2002 21 466 471 12447629 10.1007/s100670200117

[B38] Mancuso CA Sculco TP Wickiewicz TL Jones EC Robbins L Warren RF Williams-Russo P Patients’ expectations of knee surgery J Bone Joint Surg Am 2001 83-A 1005 1012 11451969 10.2106/00004623-200107000-00005

[B39] Williams O Fitzpatrick R Hajat S Reeves BC Stimpson A Morris RW Murray DW Rigge M Gregg PJ Mortality, morbidity, and 1-year outcomes of primary elective total hip arthroplasty J Arthroplasty 2002 17 165 171 11847614 10.1054/arth.2002.29389

[B40] Jamison RN Ross MJ Hoopman P Griffin F Levy J Daly M Schaffer JL Assessment of postoperative pain management: patient satisfaction and perceived helpfulness Clin J Pain 1997 13 229 236 9303255 10.1097/00002508-199709000-00008

[B41] Lingard EA Katz JN Wright EA Sledge CB Predicting the outcome of total knee arthroplasty J Bone Joint Surg Am 2004 86-A 2179 2186 15466726 10.2106/00004623-200410000-00008

[B42] Fortin PR Penrod JR Clarke AE St-Pierre Y Joseph L Belisle P Liang MH Ferland D Philips CB Mahomed N Tanzer M Sledge C Fossel AH Katz JN Timing of total joint replacement affects clinical outcomes among patients with osteoarthritis of the hip or knee Arthritis Rheum 2002 46 3327 3330 12483739 10.1002/art.10631

[B43] Guyatt GH Osoba D Wu AW Wyrwich KW Norman GR Methods to explain the clinical significance of health status measures Mayo Clin Proc 2002 77 371 383 11936935 10.4065/77.4.371

[B44] Escobar A Quintana JM Bilbao A Arostegui I Lafuente I Vidaurreta I Responsiveness and clinically important differences for the WOMAC and SF-36 after total knee replacement Osteoarthritis Cartilage 2007 15 273 280 17052924 10.1016/j.joca.2006.09.001

[B45] Quintana JM Escobar A Bilbao A Arostegui I Lafuente I Vidaurreta I Responsiveness and clinically important differences for the WOMAC and SF-36 after hip joint replacement Osteoarthritis Cartilage 2005 13 1076 1083 16154777 10.1016/j.joca.2005.06.012

[B46] Hanley JA McNeil BJ The meaning and use of the area under a receiver operating characteristic (ROC) curve Radiology 1982 143 29 36 7063747 10.1148/radiology.143.1.7063747

[B47] Judge A Cooper C Arden NK Williams S Hobbs N Dixon D Gunther KP Dreinhoefer K Dieppe PA Pre-operative expectation predicts 12-month post-operative outcome among patients undergoing primary total hip replacement in European orthopaedic centres Osteoarthritis Cartilage 2011 19 659 667 21447395 10.1016/j.joca.2011.03.009

[B48] Iversen MD Daltroy LH Fossel AH Katz JN The prognostic importance of patient pre-operative expectations of surgery for lumbar spinal stenosis Patient Educ Couns 1998 34 169 178 9731176 10.1016/s0738-3991(97)00109-2

[B49] Mancuso CA Sculco TP Salvati EA Patients with poor preoperative functional status have high expectations of total hip arthroplasty J Arthroplasty 2003 18 872 878 14566742 10.1016/s0883-5403(03)00276-6

[B50] McHugh GA Luker KA Individuals’ expectations and challenges following total hip replacement: a qualitative study Disabil Rehabil 2012 34 1351 1357 22233116 10.3109/09638288.2011.644022

[B51] Doherty M Dieppe P The "placebo" response in osteoarthritis and its implications for clinical practice Osteoarthritis Cartilage 2009 17 1255 1262 19410027 10.1016/j.joca.2009.03.023

[B52] Lingard EA Sledge CB Learmonth ID Patient expectations regarding total knee arthroplasty: differences among the United States, United kingdom, and Australia J Bone Joint Surg Am 2006 88 1201 1207 16757751 10.2106/JBJS.E.00147

[B53] Arden NK Kiran A Judge A Biant LC Javaid MK Murray DW Carr AJ Cooper C Field RE What is a good patient reported outcome after total hip replacement? Osteoarthritis Cartilage 2011 19 155 162 20951814 10.1016/j.joca.2010.10.004

[B54] Escobar A Gonzalez M Quintana JM Vrotsou K Bilbao A Herrera-Espineira C Garcia-Perez L Aizpuru F Sarasqueta C Patient acceptable symptom state and OMERACT-OARSI set of responder criteria in joint replacement. Identification of cut-off values Osteoarthritis Cartilage 2012 20 87 92 22155074 10.1016/j.joca.2011.11.007

[B55] Kvien TK Heiberg T Hagen KB Minimal clinically important improvement/difference (MCII/MCID) and patient acceptable symptom state (PASS): what do these concepts mean? Ann Rheum Dis 2007 66 Suppl 3 iii40 iii41 17934093 10.1136/ard.2007.079798 PMC2095292

[B56] Revicki D Hays RD Cella D Sloan J Recommended methods for determining responsiveness and minimally important differences for patient-reported outcomes J Clin Epidemiol 2008 61 102 109 18177782 10.1016/j.jclinepi.2007.03.012

[B57] Tubach F Ravaud P Baron G Falissard B Logeart I Bellamy N Bombardier C Felson D Hochberg M Van der Heije D Dougados M Evaluation of clinically relevant changes in patient reported outcomes in knee and hip osteoarthritis: the minimal clinically important improvement Ann Rheum Dis 2005 64 29 33 15208174 10.1136/ard.2004.022905 PMC1755212

